# Evaluation of the malaria rapid diagnostic test VIKIA malaria Ag Pf/Pan™ in endemic and non-endemic settings

**DOI:** 10.1186/1475-2875-12-188

**Published:** 2013-06-06

**Authors:** Daniel Eibach, Boubacar Traore, Mourad Bouchrik, Boubacar Coulibaly, Nianégué Coulibaly, Fanta Siby, Guillaume Bonnot, Anne-Lise Bienvenu, Stéphane Picot

**Affiliations:** 1 Institut de Parasitologie et de Mycologie Médicale, Faculty of Medicine, Hospices Civils de Lyon, Lyon, France; 2 Centre de Santé de Référence, Commune VI, Bamako, Mali; 3 Laboratoire de Parasitology & Mycology, Faculty of Medicine, Hôpital Militaire Mohamed V, Rabat, Morocco; 4 Direction Régionale de la Santé, Ministry of Health, Bamako, Mali; 5 Malaria Research Unit, CNRS UMR 5246, University Lyon 1, Lyon, France

**Keywords:** Malaria, Rapid diagnostic test, Diagnosis, Mali, Sensitivity/specificity

## Abstract

**Background:**

Malaria rapid diagnostic tests (RDTs) are a useful tool in endemic malaria countries, where light microscopy is not feasible. In non-endemic countries they can be used as complementary tests to provide timely results in case of microscopy inexperience. This study aims to compare the new VIKIA Malaria Ag Pf/Pan™ RDT with PCR-corrected microscopy results and the commonly used CareStart™ RDT to diagnose falciparum and non-falciparum malaria in the endemic setting of Bamako, Mali and the non-endemic setting of Lyon, France.

**Methods:**

Blood samples were collected during a 12-months and six-months period in 2011 from patients suspected to have malaria in Lyon and Bamako respectively. The samples were examined by light microscopy, the VIKIA Malaria Ag Pf/Pan™ test and in Bamako additionally with the CareStart™ RDT. Discordant results were corrected by real-time PCR. Sensitivity, specificity, positive predictive value and negative predictive value were used to evaluate test performance.

**Results:**

Samples of 877 patients from both sites were included. The VIKIA Malaria Ag Pf/Pan™ had a sensitivity of 98% and 96% for *Plasmodium falciparum* in Lyon and Bamako, respectively, performing similar to PCR-corrected microscopy.

**Conclusions:**

The VIKIA Malaria Ag Pf/Pan™ performs similar to PCR-corrected microscopy for the detection of *P*. *falciparum*, making it a valuable tool in malaria endemic and non-endemic regions.

## Background

Malaria diagnostic plays a key role in malaria control and elimination programmes in order to avoid unnecessary anti-malarial therapy, to prevent drug resistance and to enhance case finding. The World Health Organization (WHO) estimated that in the African region, approximately one half of suspected malaria cases received parasitological confirmation [1]. The number of courses of artemisinin combination therapy (ACT) still exceeds the total number of malaria diagnostic tests by a factor of 2, indicating that many patients receive ACT without confirmatory diagnosis [1]. The Test-Treat-Track (T3) initiative, recently launched by the WHO, is a clear indication that malaria management should be based on evidences. It is well documented that treatment based on symptoms results in overtreatment of patients who do not suffer from malaria [2]. Although clinical diagnosis is imprecise, it remains the basis of therapeutic care for too many febrile patients in malaria endemic areas, where laboratory support is often out of reach or negative slide results are disregarded. As a consequence, serious non-malarial infections are missed, drugs are wasted, resistance is increasing, and the cost effectiveness of the diagnostic tests is reduced [3-5].

The commonly accepted gold standard diagnostic method for detecting malaria was, for decades, microscopic reading of Giemsa-stained thick and thin blood films. Microscopy constrains in malaria endemic regions are the need for skilled laboratory technicians, good quality reagents and well-maintained microscopes as well as its time consumption [6]. Even in developed and non-endemic countries, expert malaria microscopists are scarce and impaired microscopy-based diagnosis, especially for non-falciparum species, in hospital laboratories is common [7-9]. Most laboratories in non-endemic countries lack sufficient samples to build-up and maintain microscopic expertise.

Malaria rapid diagnostic tests (RDTs) are an alternative diagnostic method for endemic regions, where microscopy has not been implemented, as well as for non-endemic countries, where they are able complement microscopy in screening febrile travellers. The first commercial RDT was marketed in 1994, and more than 200 devices from more than 60 manufacturers are now available in the world market. Some of them were successfully evaluated by international organizations [10-12]. Several of these RDTs showed high efficiency for the diagnosis of malaria in different conditions. One of the drawbacks of these devices is the quality of tests compared to microscopy and their practicability for local staff in remote areas [13]. In order to evaluate the performance of a new device, the VIKIA Malaria Ag Pf/Pan™ RDT, a prospective study was conducted both in an endemic area (Bamako, Mali) and a non-endemic area (Lyon, France). This RDT was during the same period, evaluated in field condition by members of the anti-malarial drug-resistance network in Cambodia [14] and showed good performances. The secondary objective of the study was to compare data obtained from technicians working in a general health care centre located in an endemic area, daily exposed to a high number of malaria diagnosis, to those obtained by technicians from an European University Hospital where malaria is restricted to two imported case per week, on a yearly basis mean. The sensitivity and specificity of VIKIA Malaria Ag Pf/Pan™ were compared with PCR-corrected microscopy results and the commonly used CareStart™ RDT in Bamako. Both RDTs are detecting falciparum and non-falciparum malaria. The VIKIA Malaria Ag Pf/Pan™ is using HRP2/Aldolase antigens while the CareStart™ is using HRP2/pLDH antigens.

## Methods

### Study design in Lyon

The first part of the study was performed during a 12-months period from January to December 2011 in the non-endemic setting of Lyon, France. Study site was the Parasitology Department of the Lyon University Hospital, were symptomatic travellers, returning from malaria endemic countries, are routinely checked for malaria. Blood samples were collected from febrile patients suspected of malaria due to recent history of travel abroad. Malaria diagnosis was carried out with Giemsa-stained thick and thin blood smears and real time PCR was performed as previously described [15]. No specific blood samples were collected for the RDT study, and data obtained with RDTs were not used for the diagnosis or treatment of patients.

### Study design in Bamako

The second part of the study was conducted between July and December 2011, in a level two general health centre (Centre de Santé de Référence de la commune VI), where minimal laboratory equipment is available, in the city of Bamako, Mali. This structure is located in a district area of approximately 800,000 inhabitants, with limited access to a higher level hospital facility. Malaria is hyperendemic in the peripheral villages, mesoendemic in the periurban area and hypoendemic in the city of Bamako [16]. Malaria is transmitted between June and December. This unstable transmission results in a non-immune population and a risk for all age groups for malarial disease. *Plasmodium falciparum* accounts for 95% of all malaria infections at the country level. Inoculation rates vary between 30 and 50 infective bites per human per transmission season in the periurban area.

All patients presenting with a temperature >37.5°C and with suspected malaria were included into the study after informed consent was obtained. Exclusion criteria were symptoms associated with signs of severe malaria according to the definitions of the WHO [17], and absence of consent from the patient or parents. Local technicians collected finger-prick samples for thick and thin blood films. The same finger-prick blood sample was used to carry out the VIKIA Malaria Ag Pf/Pan™ and CareStart™ RDTs. In addition, two drops of blood were spotted onto filter paper, individually stored in a plastic bag and sent to the Parasitology Department of the Lyon University Hospital for PCR correction. In all cases the diagnosis of malaria was confirmed or ruled out using microscopic examination of blood smears. Only results from microscopy were communicated to the patient and medical doctor for subsequent treatment if necessary. Data obtained from the two RDTs were not used for the diagnosis of the current disease. For each participant case report forms were completed, which were supervised by the local investigator, and controlled by the principal investigator. All discrepancies were resolved by consensus.

### Microscopy

Thick and thin films were prepared on a single slide. After thin films were fixed in methanol and air-dried, slides were stained in 10% Giemsa solution for 15 min. The parasitaemia was recorded as number of asexual parasites counted per 200 white blood cells present in the thick smear. The thick-film was considered to be negative, if no parasite had been found in 100 fields at 1,000 × magnification. All the thick and thin blood smears were read immediately by two local investigators in both sites, and secondly read by an expert at the Parasitology Department of the Lyon University Hospital, as a quality control. All microscopists were blinded to the results of the RDTs.

### Rapid diagnostic tests

The VIKIA Malaria Ag Pf/Pan™ test was performed by medical or pharmaceutical students at the Lyon study site, and by local community health workers at the Bamako study site, immediately after the samples were collected. The Lyon study site performed only the VIKIA Malaria Ag Pf/Pan™ (IMAccess, Lyon, France) test, while in Bamako the CareStart Malaria™ (AccessBio, USA), a commonly used RDT in Mali, was used as an additional control. Both tests were used according to manufacturer’s recommendation (lot numbers: RD_MA2_110527 (VIKIA Malaria Ag Pf/Pan™), G21MR (CareStart Malaria™)). The VIKIA Malaria Ag Pf/Pan™ test was read at different time points (15, 20, 30, 60 minutes), while the CareStart Malaria™ test was read after 20 minutes as recommended. Community health workers in Bamako and medical and pharmaceutical students in Lyon were trained by the same investigator to use both tests according to standard operating procedures. Each staff member was qualified for the global study protocol and the specific SOPs before the start of the study. An external quality monitoring in Bamako was conducted twice during the study. Study quality improvements were added to SOPs when deficiencies were identified.

### DNA extraction and amplification

All discordant results between microscopy and the two RDTs were resolved by PCR and test characteristics were recalculated according to the PCR-corrected results. DNA of blood samples from the Lyon study site was extracted using a spin-column procedure (Qiagen, Hilden, Germany). DNA from blood spots on filter papers from the Bamako study site was extracted using Instagene Matrix resin™ (Bio-Rad, Marnes la Coquette, France) [15]. Species-specific primers targeting the 18s rRNA were used to identify the parasite species, using LightCycler (Roche, Switzerland) real-time PCR [15]. The real-time PCR was submitted to a quality assurance process, including a proficiency panel provided by two independent laboratories, twice a year.

### Data analysis

The data collected were computerized using Excel program (Microsoft, USA). Statistical analysis was performed with Stata 12 (Statacorp, USA). Sensitivity was calculated as the proportion of positive test results obtained among samples containing malaria parasites as identified by PCR-corrected microscopy. Specificity was calculated as the proportion of negative test results among samples negative in PCR-corrected microscopy. Positive and negative predictive values were obtained by the proportion of true-positive results among all positive samples and the proportion of true negative results among all negative samples, respectively. Sensitivity, specificity, positive and negative predictive values were determined for both RDTs at all reading time points. A 95% confidence interval (95% CI) was given for each parameter. Samples with mixed infections were not considered in the test performance calculations. Proportions were compared using the McNemar test with p-values <0.05 considered as being significant.

### Ethical approval

Permission to perform the study was obtained from the Ministry of Health in Bamako, Mali. Informed written consent was taken from all study participants and caregivers of all children under 18 at the Bamako study site.

## Results

155 patients were included at the Parasitology Department of the Lyon University Hospital. PCR-corrected microscopy revealed 69 (44.5%) malaria positive blood samples. The majority of patients were of African ethnicity (68%), 19 patients were Caucasian (28%) and the ethnicity was unknown for three patients (4%). According to the information given by the patients, anti-malarial chemoprophylaxis was regularly taken by 32% (n=22). Most of malaria infected travellers returned from West Africa (n=54; 78%), followed by Central Africa (n=7; 10%), East Africa (n=3; 4%), India (n=2; 3%), South-East Asia (n=2; 3%) and Central Asia (n=1; 1%). *Plasmodium falciparum* was the most frequently detected species (n=49; 71%) followed by *Plasmodium ovale* (n=9; 13%), *Plasmodium vivax* (n=5; 7%) and *Plasmodium malariae* (n= 1; 1%). Mixed infections were detected in five cases (7.0%). A parasitaemia lower than 1,000 parasites/μl was observed in 16 patients (23%), a high parasitaemia above 100,000 parasites/μl was detected in 5 patients (7%).

*Plasmodium falciparum* diagnosis was confirmed in 48/49 patients and for 5/5 patients with mixed infections (Table 1), when reading the VIKIA Malaria Ag Pf/Pan™ after 30 minutes, giving a sensitivity of 98.0% (95% CI 87.8–99.9) (Table 2). Malaria diagnosis for non-falciparum species was confirmed in 8/15 patients after 15 minutes, increasing to 9/15 patients (sensitivity=60.0%; 95% CI 32.9–82.5; specificity=100%; 95% CI 96.6–100.0) when the test was read between 20 and 60 minutes. For non-falciparum species other than *P*. *ovale* the sensitivity increases to 100% (95% CI 51.7–100.0). Four negative samples and two *P*. *ovale* samples were false positive for *P*. *falciparum* after 15 minutes, increasing to three *P*. *ovale* samples positive for *P*. *falciparum* after 20 minutes. False negative results for all Plasmodium species decreased from seven to four when readings were performed at 15 and 30 minutes respectively. The test results did not differ regarding the level of parasitaemia. Differences between the VIKIA Malaria Ag Pf/Pan™ and corrected microscopy were not significant for the detection of *P*. *falciparum* (p=0.07), but significant for the detection of non-falciparum species (p=0.03).

**Table 1:** **Results of the VIKIA Malaria Ag Pf**/**Pan**™ **RDT performed in Lyon**

**PCR**-**corrected microscopy species identification**	**VIKIA Malaria Ag Pf/Pan™**											
	**15 minutes**				**20 minutes**				**30 minutes**			
	**Pf**	**Pan**	**Pf+Pan**	**no**	**Pf**	**Pan**	**Pf+Pan**	**no**	**Pf**	**Pan**	**Pf+Pan**	**no**
*P. falcipatum* (n=49)	11	0	36	2	11	0	36	2	12	0	36	1
*P. vivax* (n= 5)	0	4	0	1	0	5	0	0	0	5	0	0
*P. ovale* (n=9)	2	3	0	4	2	3	1	3	2	3	1	3
*P. malariae* (n=1)	0	1	0	0	0	1	0	0	0	1	0	0
*P. ovale* + *P. falciparum* (n=3)	0	0	3	0	0	0	3	0	0	0	3	0
*P. malariae*+ *P. falciparum* (n=2)	0	0	2	0	0	0	2	0	0	0	2	0
Negative (n=86)	2	0	2	82	2	0	2	82	2	0	2	82

**Table 2:** **Diagnostic performances of the Care start**™ **and VIKIA Malaria Ag Pf**/**Pan**™ **test** (**reading time**: **30 minutes**) **for the detection of *P***. ***falciparum *and non**-**falciparum species in Lyon and Bamako**

	**Care Start Malaria™**		**VIKIA Malaria Ag Pf/Pan™**	
	**Pf or mixed**	**non**-**Pf**	**Pf or mixed**	**non**-**Pf**
**Lyon**				
Sensitivity % (95% CI)			98.0	60.0
			(87.8–99.9)	(32.9–82.5)
Specificity % (95% CI)			93.1	100.0
			(85.8–96.9)	(96.6–100.0)
PPV % (95% CI)			87.3	100.0
			(74.9–94.3)	(62.9–100.0)
NPV % (95% CI)			98.9	95.7
			(93.4–99.9)	(90.6–98.3)
**Bamako**				
Sensitivity % (95% CI)	96.2	60.0	94.6	100
	(90.8–98.6)	(17.0–92.7)	(88.8–97.6)	(46.3–100.0)
Specificity % (95% CI)	97.7	99.4	98.0	99.2
	(96.0–98.7)	(98.5–99.8)	(96.4–98.9)	(98.1–99.7)
PPV % (95% CI)	89.9	42.9	91.1	45.5
	(83.4–94.2)	(11.8–79.8)	(84.7–95.1)	(18.1–75.4)
NPV % (95% CI)	99.1	99.7	98.8	100.0
	(97.9–99.7)	(98.9–100.0)	(97.5–99.5)	(99.3–100.0)

*PPV* positive predictive value, *NPV* negative predictive value, *Pf P*. *falciparum*, *non*-*Pf* Plasmodium non-falciparum.

At the “Centre de santé de reference de la commune VI” of Bamako, 727 patients were included during the study period. The mean age was 23.5 ± 14.9 (median=21, range 1–60) and the mean body temperature was 37.8°C (Table 3). More than 90% of the patients indicated, that they received traditional or registered drugs, including antipyretics, anti-malarials and antibiotics before arriving to the health post. However, since patients’ knowledge about drugs and diseases is considered low, data collected during medical consultation were subject to caution. For patients, who were treated before admission, the quality of drugs, the dosage and the duration of treatment remained unknown.

**Table 3:** **Study population in Bamako**, **Mali**

	**Included**	**Malaria**	**Non-malaria**
Number of patients	727	127	600
Mean age ± Standard deviation	23.5 ± 14.9	20.3 ± 18.5	24.2 ± 13.9
Median age	21	17	21
Mean temperature ± Standard deviation (°C)	37.8 ± 1.7	38.8 ± 2.8	37.5 ± 1.3
Median temperature	37.6	39	37.5

135/727 (18.6%) patients were positive for malaria parasites according to PCR-corrected microscopy of thick and thin blood smears (Table 4). None of them presented clinical signs of severity. Among the 135 malaria patients, mean blood parasitaemia at admission was 3490 ± 18950 parasites/μl (median=3250). 130 patients (96.3%) presented with *P*. *falciparum* malaria, three patients (2.2%) with *P*. *ovale* and two patients (1.5%) with *P*. *malariae*. Differences in the mean age between malaria and none-malaria patients was not significant (p<0.05). The mean body temperature at admission was higher in malaria patients than in non-malaria patients (38.8 ± 2.8 vs. 37.5 ± 1.3, respectively, (p<0.05).

**Table 4:** **Results of the Care start**™ **and the VIKIA Malaria Ag Pf**/**Pan**™ **RDT in Bamako**

**PCR**-**corrected microscopy species identification**	**Care start™ Malaria**				**VIKIA Malaria Ag Pf/Pan™**							
	**20 minutes**				**15 minutes**				**20 minutes**			
	**Pf**	**Pan**	**Pf+Pan**	**no**	**Pf**	**Pan**	**Pf+Pan**	**no**	**Pf**	**Pan**	**Pf+Pan**	**no**
*P. falciparum (n=130)*	29	0	96	5	30	0	93	7	30	0	93	7
*P. ovale (n=3)*	0	2	0	1	0	3	0	0	0	3	0	0
*P. malariae (n=2)*	0	1	0	1	0	1	0	1	0	1	0	1
Negative (n=592)	13	4	1	574	10	6	2	574	10	6	2	574

Malaria diagnosis (for all species) was confirmed in 128/135 patients in both the CARE-START Malaria™ and VIKIA Malaria Ag Pf/Pan™ test after a 30 minutes reading time (Table 4). *Plasmodium falciparum* diagnosis was confirmed in 125/130 (sensitivity=96.2%; 95% CI 90.8–98.6) and 123/130 (sensitivity=94.6%; 95% CI 88.8–97.6) patients using the CARE-START Malaria™ and VIKIA Malaria Ag Pf/Pan™ test respectively (Table 2). For non-falciparum species the CARE-START Malaria™ identified 3/5 patients (sensitivity=60.0%; 95% CI 17.0–92.7) correctly, while the VIKIA Malaria Ag Pf/Pan™ identified 4/5 patients after 15 minutes and 5/5 patients (sensitivity=100.0%; 95% CI 46.3–100.0) after 30 minutes reading time. Considering the low number of non-falciparum species detected among these patients, no definitive conclusion can be drawn from these results.

The CARE-START Malaria™ test produced 18 false positive and 7 false negative results for all Plasmodium species. Similarly, 18 false positive results and 8 false negative results after 15 minutes, decreasing to seven false negative results between 30 and 60 minutes, were obtained from the VIKIA Malaria Ag Pf/Pan™ test. For both tests the number of false negative decreased to three for a parasitaemia >200 parasites/μL and to zero when parasitaemia was >1000 parasites/μL.

No significant difference in the detection of falciparum (p=0.13) and non-falciparum (p=0.5) Malaria between the two RDTs was observed. The performance of the VIKIA Malaria Ag Pf/Pan™ test was not significantly different for *P*. *falciparum* (p=0.4), but for non-falciparum species (p=0.03) compared to PCR-corrected microscopy method.

Local staffs, with limited laboratory skills, were easily trained to the use of the VIKIA Malaria Ag Pf/Pan™. No difficulties in tests readings were noted. Tests were stored at room temperature during the study period, without detected alteration; however no specific study was conducted on the test stability. Technical instructions, provided with the tests, were considered to be clear and explicit for performing the test by local users.

## Discussion

The new VIKIA Malaria Ag Pf/Pan™ RDT showed sensitivities above 94% in Mali and 98% in France with specificities above 93% for the detection of *P*. *falciparum* malaria in endemic as well as in non-endemic settings and fulfils the requirements for a useful RDT, according to WHO standards. The test performance is comparable to the commonly used CARE-START Malaria™ RDT. At the Lyon and the Bamako sites, the test showed similar results as the PCR-corrected microscopy for *P*. *falciparum* detection. The best reading time is between 20 and 30 minutes, and should not be reduced to 15 minutes in order to limit false negative results. Similar results with the VIKIA Malaria Ag Pf/Pan™ were obtained from a study in Cambodia, showing a sensitivity of 93.4% and a specificity of 98.6% for falciparum malaria [14].

A meta-analysis calculated a mean sensitivity and specificity of 95.0% and 95.2% respectively for HRP-2 based assays [18]. However comparison between published RDT trial reports is difficult due to differences in guidelines, the study population, parasitaemia levels, reference techniques and quality of RDTs after exposure to high temperatures, high humidity or substandard transport and storage conditions.

In Bamako, the study was performed under field conditions, carried out by non-expert community health workers. The conditions were representative for a typical medical reference centre in low-income countries; therefore, the data obtained with both RDTs for malaria diagnosis were entirely satisfactory.

It is known that a small number of *P*. *falciparum* parasites show deletions or mutations of the *hrp*-*2* gene, leading to false negative results with RDTs [19,20]. The *hrp*-*2* expression in false negative samples was not tested, thus the possibility that these parasites are responsible for some false negative results cannot be excluded. It has as well been suggested that anti-*hrp*-*2* antibodies in humans may explain negative test results despite significant parasitaemia [21].

A reduced sensitivity for non-falciparum species in combined hrp-2/aldolase RDTs has been noticed in many studies [22-24], with minimal data regarding the ability of RDTs to detect *P*. *ovale*, *P*. *malariae* or even *Plasmodium knowlesi*. Considering the low number of samples with non-falciparum malaria at both study sites, statistically valid knowledge has not been obtained.

This study confirms previous abundant literature showing that RDTs are not able to replace microscopy as the gold standard for malaria diagnosis in most cases. However, RDTs have a justified place in laboratories of endemic as well as non-endemic countries. Main challenges faced in non-endemic regions are a low parasite density, altered parasite morphology due to the use of chemoprophylaxis or empiric therapy and inexperience in identifying malaria parasites due to the small number of cases seen [25]. Those problems are illustrated in the high failure rate of 11.2% for the identification of *P*. *falciparum* in U.S. laboratories [8], and failure rates of 27% and 21% in Canadian and British laboratories respectively [7,9]. Therefore, RDTs can be used complementary in non-endemic countries in situation where experienced personnel is not available, in order to perform a rapid diagnosis, which can few hours later be confirmed or corrected by microscopy or PCR.

In malaria endemic countries, RDTs may contribute to rationalization of treatment of febrile illness and reduce anti-malarial drug consumption in areas where microscopy diagnosis is not available, not reliable or not performed immediately. Successful use of RDTs can be observed in Zambia, where the consumption of artemisinin-based combination therapy could be reduced as a result [26].

## Conclusion

VIKIA Malaria Ag Pf/Pan™ showed a similar level of performance than comparative RDTs in endemic and in non-endemic settings. Community health workers were as efficient as medical students in managing malaria cases using both RDTs tested. This new RDT, recently commercially available, could be used to implement efficient malaria diagnosis in low-income endemic countries or complement PCR/microscopy diagnosis in non-endemic regions.

## Competing interests

This study was supported by IMACCESS. The fund providers had no role in data collection and analysis, decision to publish, or preparation of the manuscript.

## Authors’ contributions

DE contributed to interpretation of results and drafted the manuscript. SP and ALB designed and coordinated the study and prepared the manuscript. GB was involved in the laboratory work. BT, BC, NC and FS supervised the field study. MB assisted with data entry and laboratory work. All authors read and approved the final manuscript.
